# Integrated bioinformatics analysis to explore potential therapeutic targets and drugs for small cell carcinoma of the esophagus

**DOI:** 10.3389/fbinf.2025.1495052

**Published:** 2025-01-28

**Authors:** Maofei Zhu, Yueming Chu, Qiang Yuan, Junfeng Li, Silin Chen, Lin Li

**Affiliations:** ^1^ Department of Pharmacy, The Second Clinical Medical College of North Sichuan Medical College, Nanchong, Sichuan, China; ^2^ School of Pharmacy, North Sichuan Medical College, Nanchong, China; ^3^ Department of Cardiothoracic Surgery, The Second Clinical Medical College of North Sichuan Medical College, Nanchong, China; ^4^ Department of Oncology, The Second Clinical Medical College of North Sichuan Medical College, Nanchong, China; ^5^ Nanchong Key Laboratory of Individualized Drug Therapy, Nanchong, China

**Keywords:** bioinformatics, small cell carcinoma of the esophagus, *CDK1*, *AURKA*, DGIdb, ConnectivityMAP, anti-tumor drug

## Abstract

**Background:**

Small cell carcinoma of the esophagus (SCCE) is a rare form of esophageal cancer, which also belongs to the category of neuroendocrine malignant tumors, with a low incidence but high aggressiveness, and a very poor prognosis for patients. Currently, there is a lack of unique staging and treatment guidelines for SCCE. Therefore, a deeper understanding of the therapeutic targets and the mechanisms underlying its occurrence and development is of great importance for early diagnosis, identification of potential therapeutic agents and improvement of the prognosis for patients.

**Methods:**

Firstly, the dataset of SCCE was downloaded from the GEO database and GEO2R tool was employed for the analysis of differentially expressed genes (DEGs). GO and KEGG analysis of DEGs were carried out by using the Bioinformatics and OmicStudio tools. Then, up- and down-regulated genes were intersected with the oncogenes and the tumor suppressor genes respectively, to obtain the differentially expressed onco/tumor suppressor genes in SCCE. The STRING database was employed to conduct protein-protein interaction (PPI) analysis of differentially expressed onco/tumor suppressor genes, the network was further constructed in Cytoscape, and hub genes of the network were obtained through the Cytohubba plugin. In addition, miRDB, miRwalk, Targetscan, OncomiR, starbase, Lncbase were used to predict miRNAs and lncRNAs that regulate hub genes, the ceRNA network was built based on this. Transcription factor-miRNA co-regulatory network was analyzed in the NetworkAnalyst database and embellished in Cytoscape. Finally, drugs that may target hub genes were searched through the DGIdb and ConnectivityMAP, and docking verification was performed using Schrodinger.

**Results:**

A total of 820 genes were upregulated and 716 were downregulated, of these, 54 were identified as oncogenes and 85 as tumor suppressor genes. Seven hub genes were identified from the PPI network, which were *AURKA, BIRC5*, *CDK1*, *EZH2*, *FOXM1*, *KLF4* and *UBE2C*. Furthermore, a total of 38 drugs were searched and filtered in DGIdb and ConnectivityMAP, in the molecular docking results of drugs with hub genes, the docking score of *AURKA*, *CDK1*, and *EZH2* with multiple drugs were low (<6). In addition, crizotinib with AURKA, lapatinib with CDK1, rucaparib with EZH2, rucaparib with UBE2C were the lowest energy of all molecular docking results.

**Conclusion:**

*AURKA, BIRC5*, *CDK1*, *EZH2*, *FOXM1*, *KLF4* and *UBE2C* are the hub genes of SCCE, among them, *AURKA*, *CDK1* and *EZH2* may be used as targets of multiple drugs. Crizotinib, lapatinib, and rucaparib can act on the above targets to inhibit the progression of SCCE and play a therapeutic role.

## 1 Introduction

Small cell carcinoma of the esophagus (SCCE) is a rare histological type of esophageal cancer, which belongs to the category of neuroendocrine malignant tumors, and accounts for 0.5%–5.9% of all esophageal cancers in China (Law et al., 1994; Chen et al., 2011; Li et al., 2010a; Li et al., 2010b). Some existing studies (Chen et al., 2011; Hudson et al., 2007; Ku et al., 2008) have shown that SCCE has rapid progression, high metastasis, and extremely poor prognosis. Analysis of clinical data revealed that the tumor size, invasion depth, and metastasis rate of SCCE are significantly higher than those of esophageal squamous cell carcinoma and esophageal adenocarcinoma of the same period (Cai et al., 2019). Most patients die within 2 years of diagnosis, and their median survival is only 8–13 months (Wang et al., 2018). Clinically, SCCE is mainly diagnosed by imaging examinations and endoscopic pathological tissue biopsy, and its immunohistochemistry can detect endocrine markers (ZHANG et al., 2019). The occurrence and development of SCCE is a multi-factor, multi-stage dynamic process. Its specific mechanism is still unclear, but existing studies have revealed some relevant aspects. *PTEN* is a common tumor suppressor gene in the human body. It can inhibit the *PI3K-AKT* signaling pathway to cause cells to arrest in the G1 phase or induce apoptosis of tumor cells. Zhang et al. detected the mutation of genes such as *EGFR*, *KRAS*, *PIK3CA* and *PTEN*, and found that the incidence of *PTEN* mutation in Chinese PSCCE patients is higher than that of other esophageal cancer histological subtypes (Zhang et al., 2014). In addition, the functional status of *PTEN* will also affect the tumor immune microenvironment. In terms of cell origin, SOX2 is a transcription factor that maintains the pluripotency of tumor stem cells, studies have found that in PSCCE, SCLC and embryonic esophageal tissue, *SOX2* gene is overexpressed and tumor suppressor gene *Rb1* is expressed at a low level, but in poorly differentiated squamous cell carcinoma, the opposite is true, suggesting that PSCCE may be derived from embryonic stem cells. Mutation of the *Rb1* gene is one of the early molecular changes in PSCCE (Ishida, 2017). In addition, the occurrence of SCCE is also related to DNA damage repair. Some studies have found that *PAK1* is overexpressed in PSCCE and is positively correlated with the DNA damage marker *γH2AX*, suggesting that *PAK1* may be involved in the DNA damage/repair process of PSCCE and promote the invasion of SCCE cells. Moreover, overactivation of *PAK1* is closely related to tumor location, lymph node metastasis, and overall survival. The OS of patients with overactivated *PAK1* will also be significantly reduced. *PAK1* has the potential to become a direct target for the treatment of PSCCE (Gan et al., 2015). Through the study of genomic expression profiles, it was found that mutations in *TP53*, *RB1* and *NOTCH* family are widely present in PSCCE and SCLC, while mutations in *PDE3A*, *CBLN3* and *PTPRM3* genes have never been reported in esophageal cancer. Mutations in these three genes may be specific molecular markers in the development of PSCCE. Changes in Wnt pathway and NOTCH signaling pathway are also important events in the development of SCCE (Wang et al., 2018). In recent years, studies on the tumor immune microenvironment have also found that the immune checkpoint receptor *TIGIT* and its ligand *CD155* of SCCE are widely upregulated in PSCCE and are significantly related to distant metastasis, Ki-67 index, prognosis (Zhao K. et al., 2020).

Due to the low prevalence of SCCE, there is a lack of clinically relevant studies, usually retrospective studies or a few case-report studies, and there is no unique staging and grading system and treatment guidelines for reference. Some clinical data analyses have shown that patients with SCCE have difficulty benefiting from surgical treatment alone, which may be related to the high metastasis rate accompanying their diagnosis (Hudson et al., 2007; Cai et al., 2019). Compared with surgery, radiotherapy and chemotherapy are more meaningful for the whole treatment of SCCE, especially for patients with extensive lesions (Hudson et al., 2007; Xiao et al., 2019), which can significantly prolong the survival of patients. In the treatment plan for patients with extensive lesions, chemotherapy should be used as the basis of the entire treatment (Song et al., 2009; Vos et al., 2011; Ding et al., 2013; Xie et al., 2015), while for patients with limited lesions, radical surgical resection combined with postoperative radiotherapy and chemotherapy are the main treatment tools (Chen et al., 2011; Zhu et al., 2014). SCCE and small cell lung cancer have many similar histological and clinical pathological features, so internationally, its staging and treatment strategies are mostly based on the staging and treatment protocol of small cell lung cancer. The treatment guidelines for small cell lung cancer are generally platinum-based, in combination with other commonly used chemotherapy agents, such as etoposide or irinotecan (Amarasena et al., 2015; Ito et al., 2021). However, chemotherapy drugs are usually accompanied with tumor resistance and their effectiveness is reduced, and there are very few studies on emerging targeted therapy and immunotherapy in SCCE. In terms of treatment, it refers to the treatment experience of small cell lung cancer and neuroendocrine tumors (Cicin et al., 2007). More experimental results are still needed to confirm their effectiveness in SCCE. In summary, exploring the occurrence and development mechanism of SCCE, clarifying its tumor biological characteristics at different stages and its prognostic factors are of great significance for finding new drugs to treat SCCE or overcome chemotherapy resistance and then formulate the optimal comprehensive treatment strategy.

Since SCCE is extremely invasive, progresses rapidly, and has limited treatment options, early diagnosis and early treatment are essential. Many databases now share disease-related microarray data, which can identify gene expression profiles under different disease states. Biomarkers of a specific disease are often expressed differently in different physiological and pathological states, especially in disease states, which are extremely different from normal physiological states. Therefore, finding and evaluating these genes based on expression profiles may be an effective way for diagnosis and monitoring the prognosis of the disease (Huang et al., 2010). In addition, taking corresponding measures against hub genes in SCCE is a powerful method to manage and alleviate disease prognosis. Therefore, this study aims to mine specific markers for SCCE through gene expression data in the database, and to find suitable candidate drugs for hub genes through systems biology methods.

## 2 Materials and methods

### 2.1 Acquisition of differentially expressed genes in SCCE

Searching for “Small cell carcinoma of the esophagus” in the Gene Expression Omnibus (GEO) database, only one dataset, GSE111299, was sequenced from the genomes of patients with SCCE, which was eligible to be screened in this study (Liu et al., 2018). The platform tool GEO2R was used for online analysis, the GPL570 platform was selected and the samples were grouped into “SCCE” and “ctrl” according to their source, with three samples each. After online analysis, a complete table of expression of genes was obtained. The conditions for screening differentially expressed genes (DEGs) were: |log2FC| ≥ 2, P. Value < 0.01, removing the blanks and duplicates of “Gene. Symbol”, if there is more than one expression value for a gene, the maximum value is taken as the final value. Finally, the list of DEGs was obtained, the volcano map and heat map were performed using the OmicStudio tools (https://www.omicstudio.cn/tool) (Lyu et al., 2023).

### 2.2 GO and KEGG enrichment analysis of DEGs in SCCE

In order to elucidate the biological functions and pathways associated with the identified DEGs, a functional enrichment analysis was conducted using the Gene Ontology (GO) database. Additionally, the Kyoto Encyclopedia of Genes and Genomes database (KEGG) database was employed to examine the signaling pathways with significantly enriched differential genes. Enrichment analysis was performed by https://www.bioinformatics.com.cn, an online platform designed for analysis and visualization of data (Tang et al., 2023). P < 0.05 in the enrichment results is considered significant. The GO enrichment analysis diagram was automatically generated and visualized in the bioinformatics platform. The top10 signal pathways were selected based on the P value of the KEGG enrichment analysis results and visualized in the OmicStudio tools.

### 2.3 Onco and tumor suppressor genes and their PPI network in SCCE

Human oncogenes and tumor suppressor genes (TSGs) were downloaded from oncogene database (https://bioinfo-minzhao.org/ongene/) (Liu et al., 2017) and TUMOR SUPPRESSOR GENE DATABASE (https://bioinfo.uth.edu/TSGene/index.html) (Zhao et al., 2016), respectively. Then VENNY2.1.0 was used to intersect oncogenes with upregulated genes and TSGs with downregulated genes, to gain upregulated oncogenes and downregulated TSGs in SCCE. Next, the PPI network of oncogenes and TSGs in SCCE was built and visualized in a search tool for the retrieval of interacting genes/proteins (STRING) (https://cn.string-db.org/) version 12.0 and Cytoscape (https://cytoscape.org/) version 3.10.0.

### 2.4 Identification of hub genes from PPI network

In order to further screen hub genes in SCCE, the cytohubba plugin (https://apps.cytoscape.org/apps/cytohubba) was used in the cytoscape and its’ 12 topological analysis methods (MCC, DMNC, MNC, Degree, EPC, BottleNeck, EcCentricity, Closeness, Radiality, Betweenness, Stress, ClusteringCoefficient) were utilized to calculate. The top 10 genes in each method were obtained, and the genes that were in the top 10 in six or more algorithms were selected and considered to be hub genes in SCCE.

### 2.5 Survival analysis of hub genes

To further verify the obtained hub genes, we used the GEPIA database for gene expression analysis and survival analysis (Tang et al., 2017). Data from TCGA and GETx were used to first explore the expression of hub genes in ESCA, and then explore the association between survival probability and the expression of hub genes. We focused on ESCA patients and evaluated overall survival (OS) and disease free survival (DFS). Plots with a median cutoff of 50% and hazard ratios with 95% confidence intervals were generated, with statistical significance both set at P < 0.05.

### 2.6 Construction of ceRNA and TF-miRNA co-regulatory network

In order to explore the regulatory network related to hub genes and better clarify the role of hub genes, the upstream miRNAs of seven hub genes were predicted through three databases: miRwalk (http://mirwalk.umm.uni-heidelberg.de/), miRDB (https://mirdb.org/), and Targetscan (https://www.targetscan.org/vert_80/), and the overlapping data of the three were taken; in the OncomiR (https://oncomir.org/oncomir) database, select ESCA as a cancer type to obtain relevant miRNAs. The significant miRNAs that appear in the four databases at the same time were considered to be the miRNAs that regulate hub genes. The filtered miRNAs were entered into starbase (starBase or ENCORI: Decoding the Encyclopedia of RNA Interactomes (rnasysu.com)) and Lncbase (https://dianalab.e-ce.uth.gr/html/diana/web/index.php?r=lncbasev2) for search, the results obtained were paired one by one with miRNA-lncRNA into a set of data, duplicates were removed. After sorting the correlation data of hub genes, miRNAs, and lncRNAs, the ceRNA network visualization analysis was performed in Cytoscape.

NetworkAnalyst (https://www.networkanalyst.ca/) version 3.0 can embed the target gene into the biological network of 15 different databases and extract the genes/proteins, miRNAs, drugs, chemicals or diseases that are most closely related to the uploaded genes (seed genes) (Zhou et al., 2019). Enter the hub genes’ name in the NetworkAnalyst, and select TF-miRNA coregulatory interaction database, the TF-miRNA co-regulatory network related to the hub genes was obtained after analysis, then visualized in Cytoscape.

### 2.7 Construction of GeneMANIA-based functional association network

To further identify genes related to the functions of hub genes, we used a novel approach from the GeneMANIA database to find additional 10 genes with the strongest associations for each hub gene (Rajadnya et al., 2024). This analysis selected three key parameters, namely, co-expression, gene interactions, and physical interactions, to improve the accuracy of target identification. Subsequently, all newly discovered genes were merged with the initially obtained hub genes to create a GMFA-based SCCE target expansion database network. Finally, GO and KEGG enrichment analysis were performed on these expanded genes.

### 2.8 Screening for drug candidates

DGIdb (https://www.dgidb.org/) version 5.0.6 is an open source search engine for drug-gene interactions and druggable genomes (Cannon et al., 2024). In order to find potential therapeutic drugs for SCCE, a search was conducted in the DGIdb. Hub genes were used as input to predict the drugs targeting them, anti-tumor drugs approved by the FDA were screened as drug candidates in this study.

Based on information about differences in gene expression, the ConnectivityMAP (https://clue.io/) uses a pattern matching algorithms to infer whether there is a functional correlation between drugs, genes and diseases. Its query application can be used to find positive and negative connections between the gene expression features of interest and all features in ConnectivityMAP (Lamb, 2007). The upregulated oncogenes and downregulated TSGs were input and queried in the Query tool of ConnectivityMAP. In the results, a negative value of “raw_cs” indicates that the compound may be effective against the disease. Therefore, the top 10 compounds were selected as drug candidates based on the sorting and screening of FDA-approved anti-tumor drugs.

### 2.9 Molecular docking

The structures of drug candidates were sourced from the PubChem database (https://pubchem.ncbi.nlm.nih.gov/). Firstly, imported these drug structures into the Schrodinger software version 2024.01 to establish a database, following hydrogenation, structural optimization, and energy minimization, the resulting ligand molecules were saved for use in molecular docking. The target protein *AURKA* (PDB ID: 4UYN), *BIRC5* (PDB ID: 7LBQ), *CDK1* (PDB ID: 6GU2), *EZH2* (PDB ID: 5HYN), *FOXM1* (PDB ID: 3G73), *KLF4* (PDB ID: 6VTX), *UBE2C* (PDB ID: 4YII) structures were sourced from the RCSB database (https://www.rcsb.org/), then protein structures were processed on the Maestro (version 2024.01) platform, and the proteins were processed using the ProteinPreparationWizard of Schrodinger. Finally, the proteins were minimized and the geometry structures were optimized. The receptor underwent pre-processing, optimization, and minimization (with the OPLS4 force field being utilized for constrained minimization). The LigPrep module’s default settings were followed in preparing the compound structures. The constructed receptor was imported, the protein’s natural ligand was selected as the target’s active site center, and the active site position was generated in the receptor grid for screening in the Glide module. Lastly, the molecular docking was carried out using the recognized standard docking methodology (Standard Precision, SP) (Friesner et al., 2004; Rajeswari et al., 2014; Fazi et al., 2015). The Schrödinger software support was provided by Nanjing University of Chinese Medicine.

## 3 Results

### 3.1 Identification and enrichment analysis of differentially expressed genes

**FIGURE 1:**
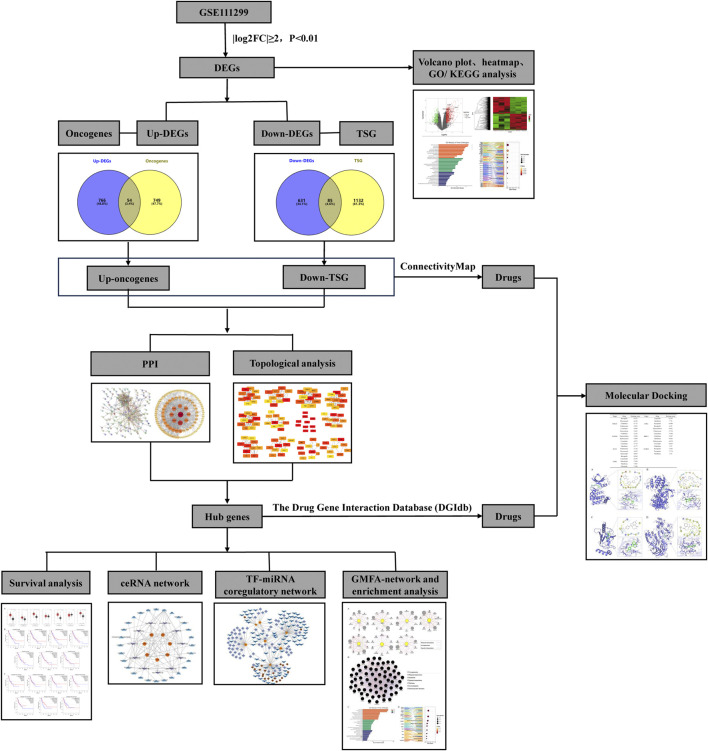
Workflow of the analytical process performed in this study.

**FIGURE 2:**
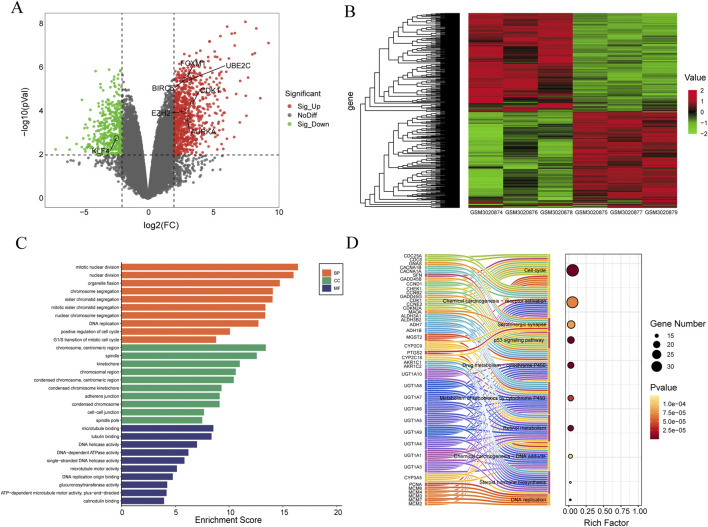
Identification and enrichment analysis of differentially expressed genes in the GSE111299 datasets. **(A)** Volcano plot, the red dots represent upregulated genes, the green dots represent downregulated genes, and the gray dots represent genes with no significant difference in expression. **(B)** Heat map of the DEGs, the left of the heat map shows clustering of the DEGs, red represents upregulated genes, and green represents downregulated genes (n = 3). **(C)** Top 10 Gene Ontology (GO) terms related to Biological Process (BP), Cellular Component (CC), and Molecular Function (MF). The length of the bars reflects the enrichment score of each GO term. **(D)** Sankey Bubble Plot of top 10 KEGG pathways and related genes. The size of the bubble represents the number of DEGs enriched in a pathway, and the color of bubble represents enrichment P-value of DEGs in each pathway, brown colors represent relative high enrichment p-values, while yellow colors represent relative low enrichment P-values. The X-axis represents rich factor of each pathway.

The process of this study is shown in Figure 1. In the GSE111299 dataset, a total of 1,536 differentially expressed genes in SCCE (|log2FC| ≥ 2 and P. Value < 0.01) were screened, 820 genes were upregulated and 716 genes were downregulated. The volcano plot shows the distribution of these DEGs (Figure 2A), and the clustering heat map of DEGs is shown in Figure 2B, GSM3020874, GSM3020876, and GSM3020878 are tumor tissues of patients with SCCE, while GSM3020875, GSM3020877, and GSM3020879 are normal tissues of the corresponding patients. It can be seen that the gene expression of tumor and normal tissues is significantly different.

To gain insight into the biological classification of the identified DEGs, Gene Ontology (GO) and Kyoto Encyclopedia of Genes and Genomes (KEGG) pathway enrichment analysis were performed. Figure 2C shows the enrichment analysis results of biological processes, which show that DEGs are particularly enriched in aspects such as mitotic nuclear division, organelle fission, chromosome segregation, etc. DEGs also significantly enriched cellular components such as chromosome, centromeric region, spindle, kinetochore. In terms of molecular functions, DEGs are mainly related to microtubule binding, tubulin binding, and DNA helicase activity. etc. Bar length represents the enrichment score of each GO term. Figure 2C shows the top 10 results for biological processes (BP), cellular components (CC), and molecular functions (MF).

In the KEGG pathway analysis, several important pathways were identified, including cell cycle, Chemical carcinogenesis-receptor activation, p53 signaling pathway, and so on. The left side of Figure 2D shows the interaction between representative genes and these 10 KEGG pathways in the form of a Sankey diagram. The size of the bubble chart circle on the right side indicates the number of differentially enriched genes in the pathway, and the color of the circle indicates the enrichment p-value of DEGs in each pathway. The darker the color, the higher the enrichment p-value. The X-axis indicates the enrichment score of each pathway.

### 3.2 Onco and tumor suppressor genes in SCCE and protein-protein interaction

**FIGURE 3:**
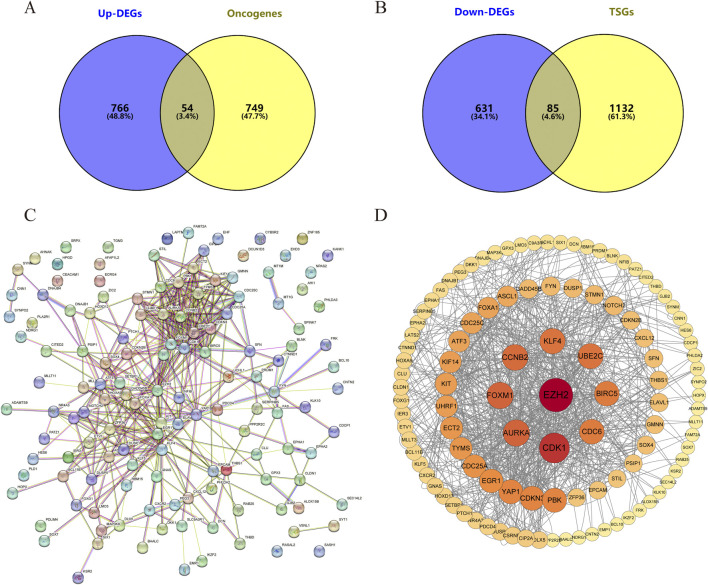
The differentially expressed oncogenes and tumor suppressor genes of small cell esophageal carcinoma and their protein-protein interactions. Intersection of **(A)** Upregulated differentially expressed genes vs. Oncogenes and **(B)** downregulated differentially expressed genes vs. Tumor suppressor gene. **(C, D)** Protein-protein interactions of differentially expressed oncogenes and TSGs. Each node represents a gene, and the colors of nodes in **(D)** indicates the significance of genes. Red colors represent relative high significance of genes, while yellow colors represent relative low degrees.

The intersections between upregulated DEGs and oncogenes and between downregulated DEGs and TSGs were analyzed using Venny 2.1.0. The results are shown in Figures 3A, B, among the 820 upregulated genes and 716 downregulated genes, there were 54 upregulated oncogenes and 85 downregulated TSGs. The protein interactions of upregulated oncogenes and downregulated TSGs showed 136 interaction nodes, 407 edges, an average node degree of 5.99, and an average local clustering coefficient of 0.411 (Figures 3C, D). The protein interaction network was visualized using Cytoscape and sorted by degree value. The larger the circle and the darker the color, the higher degree of the protein. It is in a key position in this interaction network and is the core of the entire network. It can be considered as a key target for SCCE.

### 3.3 Identification of hub genes from PPI network

**FIGURE 4:**
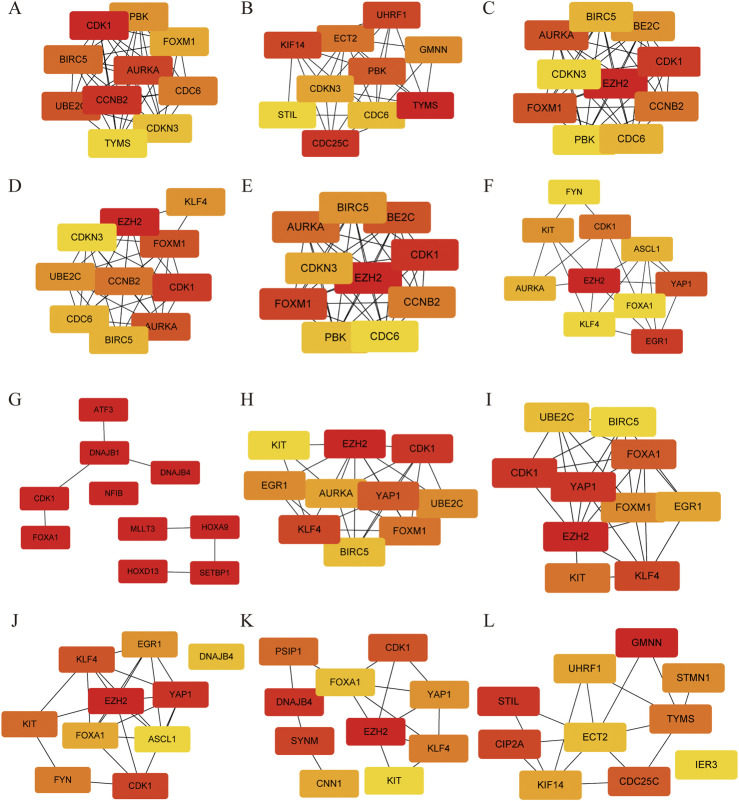
Protein-Protein interaction of hub genes using different topological methods. **(A)** MCC, **(B)** DMNC, **(C)** MNC, **(D)** Degree, **(E)** EPC, **(F)** Bottleneck, **(G)** EcCentricity, **(H)** Closeness, **(I)** Radiality, and **(J)** Betweenness, **(K)** Stress and **(L)** Clustering Coefficient. Red colors represent relative high score, while yellow colors represent relative low score.

Further analysis of the PPI network by 12 different topological analysis methods found that seven genes (*AURKA*, *BIRC5*, *CDK1*, *EZH2*, *FOXM1*, *KLF4*, *UBE2C*) appeared repeatedly at top 10 among at least 50% of the topological analysis methods (Figure 4; Table 1). They are considered to be the hub genes of SCCE, which may have certain value in the diagnosis, treatment and prognosis of SCCE. However, the effectiveness of these hub genes still needs further experimental verification.

**TABLE 1:** Hub genes identified via the different topological analysis and their functional roles.

Different topological analysis	Genes	Full name	Function
MCC, MNC, Degree, EPC, BottleNeck, Closeness	*AURKA*	Aurora Kinase A	The protein encoded by this gene is a cell cycle-regulated kinase that appears to be involved in microtubule formation and/or stabilization at the spindle pole during chromosome segregation. The encoded protein is found at the centrosome in interphase cells and at the spindle poles in mitosis
MCC, MNC, Degree, EPC, Closeness, Radiality	*BIRC5*	Baculoviral IAP Repeat Containing 5	This gene is a member of the inhibitor of apoptosis (IAP) gene family, which encode negative regulatory proteins that prevent apoptotic cell death. IAP family members usually contain multiple baculovirus IAP repeat (BIR) domains, but this gene encodes proteins with only a single BIR domain
MCC, MNC, Degree, EPC, BottleNeck, EcCentricity, Closeness, Radiality, Betweenness, Stress	*CDK1*	Cyclin-dependent kinase 1	The protein encoded by this gene is a member of the Ser/Thr protein kinase family. This protein is a catalytic subunit of the highly conserved protein kinase complex known as M-phase promoting factor (MPF), which is essential for G2/M phase transitions of eukaryotic cell cycle. Mitotic cyclins stably associate with this protein and function as regulatory subunits
MCC, MNC, Degree, EPC, BottleNeck, Closeness, Radiality, Betweenness, Stress	*EZH2*	Enhancer Of Zeste 2 Polycomb Repressive Complex 2 Subunit	This gene encodes a member of the Polycomb-group (PcG) family. PcG family members form multimeric protein complexes, which are involved in maintaining the transcriptional repressive state of genes over successive cell generations. This protein associates with the embryonic ectoderm development protein, the VAV1 oncoprotein, and the X-linked nuclear protein
MCC, MNC, Degree, EPC, Closeness, Radiality	*FOXM1*	Forkhead Box M1	The protein encoded by this gene is a transcriptional activator involved in cell proliferation. The encoded protein is phosphorylated in M phase and regulates the expression of several cell cycle genes, such as cyclin B1 and cyclin D1
Degree, EPC, Closeness, Radiality, Betweenness, Stress	*KLF4*	KLF Transcription Factor 4	This gene encodes a protein that belongs to the Kruppel family of transcription factors. The encoded zinc finger protein is required for normal development of the barrier function of skin. The encoded protein is thought to control the G1-to-S transition of the cell cycle following DNA damage by mediating the tumor suppressor gene p53
MCC, MNC, Degree, EPC, Closeness, Radiality	*UBE2C*	Ubiquitin Conjugating Enzyme E2 C	The modification of proteins with ubiquitin is an important cellular mechanism for targeting abnormal or short-lived proteins for degradation. Ubiquitination involves at least three classes of enzymes: ubiquitin-activating enzymes, ubiquitin-conjugating enzymes, and ubiquitin-protein ligases. This gene encodes a member of the E2 ubiquitin-conjugating enzyme family

### 3.4 Survival analysis of hub genes

**FIGURE 5:**
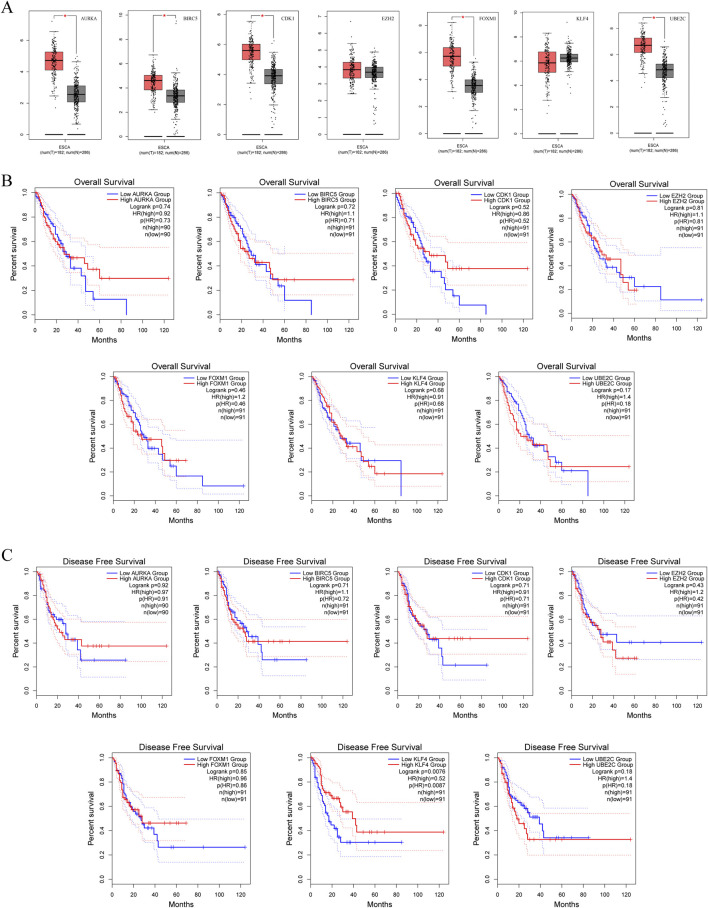
Survival analysis predicting the relationship between gene expression patterns of seven hub genes and patient survival outcomes in ESCA. **(A)** Expression of hub genes in ESCA, **(B)** Overall Survival (OS) analysis, **(C)** Disease Free Survival (DFS) analysis.

Since we lack clinical survival data for SCCE, and considering that SCCE is a subtype of esophageal cancer, we used the data of ESCA in TCGA and GETx for analysis. Using GEPIA, we first analyzed the expression of seven hub genes in ESCA and found that their expression was roughly similar to that in SCCE, and there was no statistically significant difference in the expression of *EZH2* and *KLF4* (Figure 5A). The overall survival (OS) and disease free survival (DFS) of the hub genes were then analyzed, with a median cutoff value of 50% and a 95% confidence interval for the hazard ratio. In the OS analysis, with the progression of ESCA, high expression of *BIRC5*, *EZH2*, *FOXM1*, and *UBE2C* promoted patient death, while high expression of *KLF4* prolonged patient survival (Figure 5B). In DFS analysis, high expression of *BIRC5*, *EZH2* and *UBE2C* will reduce the survival rate of patients, on the contrary, high expression of *KLF4* will significantly improve the survival rate of patients, with significant statistical significance (p (HR) = 0.0087) (Figure 5C). These results indicate that the expression differences of the hub genes are largely associated with the prognosis of ESCA patients, further suggesting that hub genes have a certain significance for the prognosis of SCCE.

### 3.5 Construction of ceRNA and TF-miRNA co-regulatory network

**FIGURE 6:**
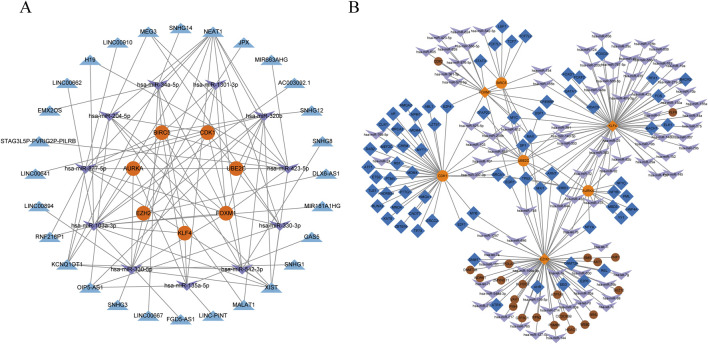
Construction of a ceRNA network and a TF-miRNA coregulatory network. **(A)** ceRNA network, the orange circles represent the hub genes, the purple arrows represent miRNAs, and the blue triangles represent lncRNAs. **(B)** TF-miRNA coregulatory network, the orange circles represent the hub genes, the purple arrows represent miRNAs, and the blue diamond represent transcription factors, the brown circles represent other related genes.

In order to better understand the hub genes and their associated regulatory mechanisms, relevant miRNAs, lncRNAs (Supplementary Figure S1), and transcription factors were predicted from the database, the ceRNA and TF-mRNA regulatory networks based on hub genes were constructed, as shown in Figure 6, including 15 miRNAs (Table 2) and 27 relevant lncRNAs. These miRNAs, lncRNAs and transcription factors may have certain regulatory capabilities for the expression or function of hub genes, which is worthy of further study.

**TABLE 2:** Differential expression analysis of the predicted miRNAs in OncomiR.

miRNA name	Cancer abbreviation	T-Test P-value	*t*-Test FDR	Upregulated in	Tumor Log2 mean expression	Normal Log2 mean expression
hsa-miR-30c-2-3p	ESCA	0.0237	0.082	Normal	4.40	5.19
hsa-miR-153-3p	ESCA	0.029	0.0975	Normal	0.74	2.06
hsa-miR-877-5p	ESCA	0.0000274	0.00129	Tumor	1.56	0.27
hsa-miR-103a-3p	ESCA	0.0000615	0.00215	Tumor	14.53	13.93
hsa-miR-1301-3p	ESCA	0.000128	0.00279	Tumor	3.11	1.58
hsa-miR-29b-1-5p	ESCA	0.000523	0.00618	Tumor	2.79	1.48
hsa-miR-204-5p	ESCA	0.00215	0.0155	Normal	0.63	3.94
hsa-miR-330-5p	ESCA	0.00545	0.0289	Tumor	4.43	3.81
hsa-miR-542-3p	ESCA	0.0076	0.0385	Tumor	7.35	6.54
hsa-miR-767-5p	ESCA	0.0107	0.0488	Tumor	3.17	0.00
hsa-miR-423-5p	ESCA	0.0113	0.0506	Tumor	6.08	5.64
hsa-miR-34a-5p	ESCA	0.0218	0.0787	Tumor	7.17	6.49
hsa-miR-320b	ESCA	0.0231	0.0816	Tumor	2.46	1.64
hsa-miR-330-3p	ESCA	0.0251	0.0861	Tumor	1.30	0.82
hsa-miR-135a-5p	ESCA	0.0347	0.113	Normal	0.87	2.30

### 3.6 Construction of GeneMANIA-based functional association network and enrichment analysis

**FIGURE 7:**
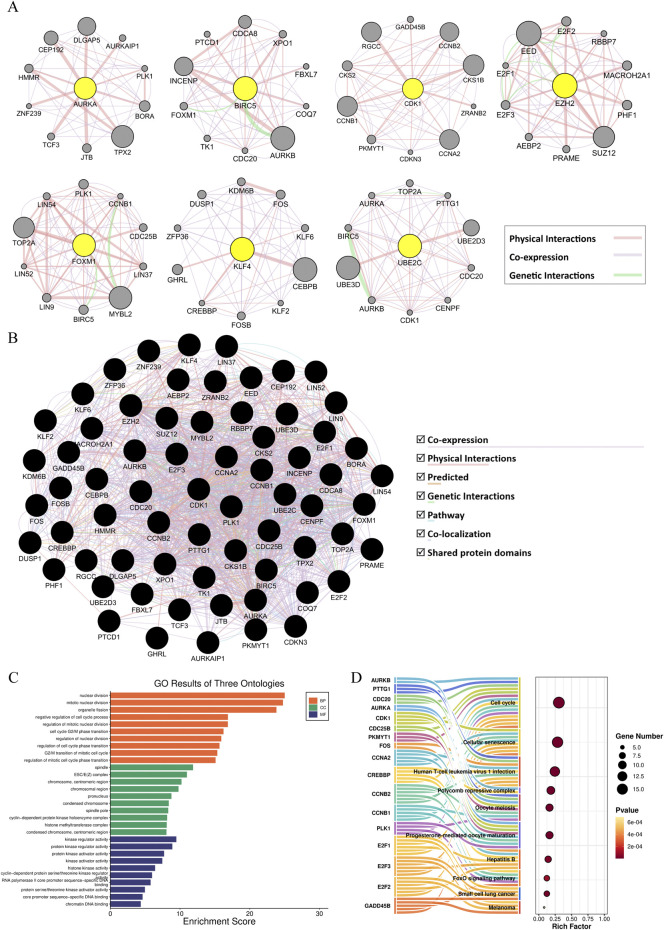
Construction of GeneMANIA-based functional association network and enrichment analysis. **(A)** GeneMANIA functional association (GMFA) network analysis illustrating functionally related genes associated with the seven hub genes of SCCE. **(B)** Functional gene network of all 67 genes related to SCCE. **(C)** Top 10 Gene Ontology (GO) terms related to Biological Process (BP), Cellular Component (CC), and Molecular Function (MF). The length of the bars reflects the enrichment score of each GO term. **(D)** Sankey Bubble Plot of top 10 KEGG pathways and related genes. The size of the bubble represents the number of DEGs enriched in a pathway, and the color of bubble represents enrichment P-value of DEGs in each pathway, brown colors represent relative high enrichment p-values, while yellow colors represent relative low enrichment P-values. The X-axis represents rich factor of each pathway.

To further explore genes associated with hub gene functions, a novel analysis method of GeneMANIA was used to identify 10 additional genes for each hub gene (Figure 7A), which were organized into the GMFA extended database (GMFA-ED), which contains 67 genes after removing duplicates. The GMFA method integrates co-expression, genetic interaction, and physical interaction parameters to capture various genes that are strongly associated with hub genes and form a network diagram, as shown in Figure 7B. GO and KEGG enrichment analysis of GMFA-ED found that these genes were significantly enriched in nuclear division, mitotic nuclear division, organelle fission and the regulation of cell cycle in BP, significantly enriched in spindle, ESC/E(Z) complex, chromosome, centromeric region in CC, and significantly enriched in kinase regulator activity, protein kinase regulator activity, protein kinase activator activity, et al. in MF (Figure 7C). The pathway enrichment results showed that, like the previous enrichment, the most significantly enriched was still Cell cycle, but more surprisingly, the “FoxO signaling pathway” and “Small cell lung cancer” were enriched (Figure 7D). Based on the similarities between small cell lung cancer and SCCE, this enrichment result confirms that these genes are likely to play important roles in SCCE.

### 3.7 Searching for drug candidates targeting hub genes

There are many databases containing information on drug action, which can be used as a basis for predicting drugs that may target certain genes. Through conditional screening, it may lead to the discovery of new drugs for the treatment of SCCE. This study used the DGIdb database to search drugs that targeting hub genes. Rucaparib for targeting *CDK1*, dabrafenib and tazemetostat hydrobromide for targeting *EZH2*, and vorinostat, omacetaxine mepesuccinate, paclitaxel, epirubicin, doxorubicin, erlotinib, dactinomycin, lapatinib, cisplatin, imatinib, irinotecan hydrochloride, plicamycin, carboplatin, lonafarnib, trastuzumab, cytarabine, arsenic trioxide, flutamide, docetaxel, INFβ-1A, romidepsin, fluorouracil for targeting *BIRC5*, and that targeted *AURKA* are paclitaxel, fluorouracil, cisplatin, entrectinib, sorafenib, pazopanib, tamoxifen, and drug targeting *KLF4* is hydroxyurea. In addition, the ConnectivityMAP can query potentially effective drugs based on DEGs, upregulated oncogenes and downregulated TSGs in SCCE were imported for query. After filtering, drug candidates were selected including etoposide, valrubicin, pralatrexate, KPT-330 (selinexor), daunorubicin, teniposide, lonafarnib, irinotecan, cladribine, and crizotinib, as shown in Table 3. All of the above drugs were considered as therapeutic drug candidates for SCCE, and their effectiveness can be further verified.

**TABLE 3:** Potential drug candidates analyzed by the DGIdb and ConnectivityMap (FDA approved anti-tumor drugs).

Databases	Genes	Drugs
DGIdb	*AURKA*	Paclitaxel, Fluorouracil, Cisplatin, Entrectinib, Sorafenib, Pazopanib, Tamoxifen
	*BIRC5*	Vorinostat, Omacetaxine mepesuccinate, Paclitaxel, Epirubicin, Doxorubicin, Erlotinib, Dactinomycin, Lapatinib, Cisplatin, Imatinib, Irinotecan hydrochloride, Plicamycin, Carboplatin, Lonafarnib, Trastuzumab, Cytarabine, Arsenic trioxide, Flutamide, Docetaxel, INFβ-1A, Romidepsin, Fluorouracil
	*CDK1*	Rucaparib
	*EZH2*	Dabrafenib, Tazemetostat hydrobromide
	*KLF4*	Hydroxyurea
ConnectivityMap	54 upregulated oncogenes and 85 downregulated TSGs	Etoposide, Valrubicin, Pralatrexate, KPT-330 (Selinexor), Daunorubicin, Teniposide, Lonafarnib, Irinotecan, Cladribine, Crizotinib

### 3.8 Molecular docking of hub genes with drug candidates

In order to ascertain the binding ability between drug candidates and hub genes, molecular docking experiments were conducted to determine the interaction between drugs and protein residues, including hydrogen bonding, π-π interactions, hydrophobic interactions, and so forth. The docking scores were then referenced to infer whether the drugs exhibited specific active effects.

**FIGURE 8:**
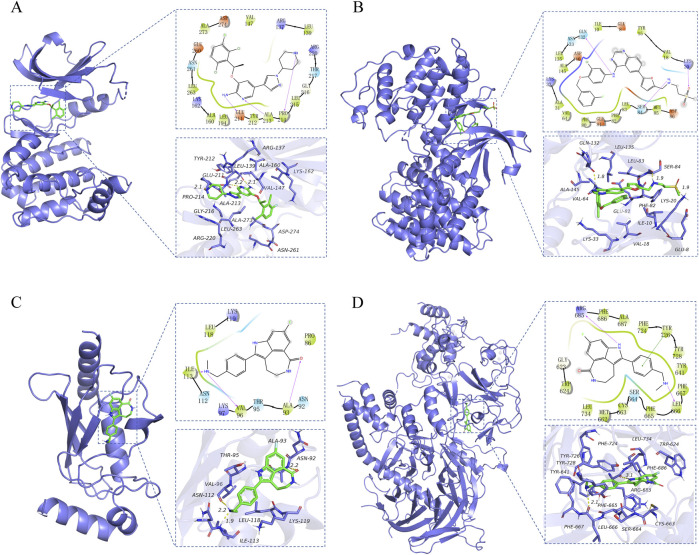
Three- and Two-dimensional presentation of molecular docking results. **(A)** *AURKA*-Crizotinib, **(B)** *CDK1*-Lapatinib, **(C)** *UBE2C*-Rucaparib, **(D)** *EZH2*-Rucaparib. The purple arrowhead represents hydrogen bond interactions, the green line with both ends presents π-π stacking.

In this study, 38 clinically approved anti-cancerous drug candidates were molecularly docked with the target proteins *AURKA*, *BIRC5*, *CDK1*, *EZH2*, *FOXM1*, *KLF4* and *UBE2C*. The molecular docking results indicated that drugs had strong binding interactions with the target proteins and a high degree of match (docking score less than −6). The top five compounds with the strongest affinity for each target are listed in Table 4, and the interactions between drugs and the highest binding affinity for each target protein (docking score less than −6) are shown in Figure 8. Based on the comprehensive results, *AURKA*, *CDK1* and *EZH2* may be used as therapeutic targets for SCCE, and the docking score of the corresponding drugs are all less than −6. In addition, the crizotinib can form hydrogen bond interactions with the GLU-211, ALA-213 and PRO-214 amino acids of the *AURKA* protein, and can also form strong hydrophobic interactions with ASN-261, LEU-263, LYS-162, etc., the compound forms van der Waals interactions with the surrounding amino acids. Lapatinib can form hydrogen bond interactions with amino acids GLN-132, SER-84 and LYS-20 of *CDK1* protein, and hydrophobic interactions with amino acids such as MET-85, ILE-10, and TYR-15, which enhance the binding ability between the two. Rucaparib is inserted deep into the *EZH2* protein pocket, fit well with the protein pocket, and form hydrogen bond interactions with the ARG-685, TYR-726 and LEU-666 amino acid and hydrophobic interactions with the surrounding hydrophobic amino acids (PHE-686, TYR-641, PHE-665, LEU-734, etc.). Similarly, rucaparib compounds are also able to form hydrogen bonding (ILE-113, VAL-96, ALA-93), hydrophobic (LEU-118, PRO-86) and other interactions with *UBE2C* proteins, and these interactions are effective in inducing the formation of stable complexes between small molecules and proteins. The above hub proteins have been fully studied, and their potential binding sites have been illustrated by researchers through molecular docking and molecular dynamics simulations, which overlap with the binding sites in the results of this study. In summary, the drug candidates are able to form different kinds of interactions such as hydrogen bonding, hydrophobic bonding, and conjugation with protein targets, these interactions can effectively help proteins and compounds to form stable complexes.

**TABLE 4:** Docking results of drug candidates with hub genes (top five respectively).

Target	Drug	Docking score	Target	Drug	Docking score
*AURKA*	Crizotinib	−8.025	*FOXM1*	Doxorubicin	−5.681
	Cladribine	−7.87		Cladribine	−5.163
	Rucaparib	−7.845		Daunorubicin	−5.044
	Pazopanib	−7.578		Hydroxyurea	−5.004
	Valrubicin	−7.35		Cytarabine	−4.872
*BIRC5*	Pralatrexate	−5.726	*KLF4*	Cytarabine	−5.722
	Cytarabine	−5.586		Valrubicin	−5.177
	Cladribine	−5.097		Pralatrexate	−5.136
	Valrubicin	−4.882		Fluorouracil	−4.959
	Hydroxyurea	−4.859		Entrectinib	−4.887
*CDK1*	Lapatinib	−8.505	*UBE2C*	Rucaparib	−6.455
	Valrubicin	−7.76		Fluorouracil	−6.336
	Pazopanib	−6.996		Cladribine	−5.773
	Rucaparib	−6.93		Pralatrexate	−5.091
	Daunorubicin	−6.891		Cytarabine	−4.996
*EZH2*	Rucaparib	−8.936			
	Lonafarnib	−8.536			
	Dabrafenib	−7.989			
	Valrubicin	−7.967			
	Flutamide	−7.449			

## 4 Discussion

SCCE is a rare neuroendocrine carcinoma originating from the esophagus, studies have shown that it has a low incidence, strong invasiveness, and poor prognosis. Precisely because of its low incidence, there are few related studies, and most of them are case study reports. It is difficult to study the occurrence and development mechanism of SCCE, which leads to a single clinical treatment method and a lack of chemotherapy drugs. Therefore, this study aims to mine the transcriptome sequencing data of SCCE by bioinformatics methods, analyze the hub genes of SCCE, and then find potential therapeutic drugs, so as to establish a theoretical basis for elucidating the molecular mechanism of SCCE, exploring diagnostic and prognostic factors, and finding therapeutic drugs.

Over the past few years, with the continuous development of omics technology, researchers have analyzed the gene expression profiles of SCCE and revealed some of the possible molecular mechanisms. Wang et al. constructed a comprehensive genome map of PESC for the first time (Wang et al., 2018), They conducted whole-exome, SNP chip detection and high-depth targeted sequencing analysis on 55 patients, and found three significant but not yet reported and mutated genes *PDE3A*, *PTPRM*, *CBLN2*. Li et al. conducted genome and transcriptome analysis on 65 SCCE patient tissues and found that SCCE has *RB1* destruction mediated by multiple mechanisms (Li et al., 2021a). These sequencing analyses have identified potential therapeutic targets for SCCE from different perspectives. In addition, Okumura et al. examined miRNA expression in SCCE tumor tissues and established a cell line (TYUC-1), they found that eight miRNAs were significantly associated with tumors, and validation in the cell line revealed that downregulate MiR-625 can significantly inhibited the migration of TYUC-1, suggesting that miRNAs may also have a role in the diagnosis of SCCE (Okumura, 2015). Furthermore, studies have shown that *PAK1* (Gan et al., 2015), Wnt pathway (Chen et al., 2014), tumor immune microenvironment (Zhao Q. et al., 2020), etc. also play an important role in the development of SCCE.

In this study, data from the GEO database were used to conduct a new analysis of the gene expression results of SCCE tissue samples and normal tissue samples. The screening conditions for DEGs were: |log2FC| ≥ 2, P. Value < 0.01. A total of 1,536 DEGs were finally identified, the up- and downregulated genes were intersected with oncogenes and TSGs, respectively, to obtain 54 upregulated oncogenes and 85 downregulated TSGs. The functional effects and pathways associated with these genes were confirmed by GO and KEGG pathway enrichment analysis. The hub genes (*AURKA, BIRC5, CDK1, EZH2, FOXM1, KLF4, UBE2C*) were identified by constructing a PPI network and topological analysis methods, and other regulatory networks that interact with hub genes were analyzed through multiple databases. Small cell lung cancer and SCCE are both neuroendocrine tumors, gene expression profiling analysis found that the two have very similar mitotic and DNA repair gene expression patterns (Liu et al., 2021). Feng Wang et al.'s genome sequencing analysis and comparison of SCCE showed that SCCE is very similar to esophageal squamous cell carcinoma, even more similar than SCLC (Wang et al., 2018). Therefore, referring to the relevant studies on small cell lung cancer and esophageal squamous cell carcinoma, we may be able to obtain effective information about SCCE. Among the hub genes, *CDK1* encodes protein which is a member of the Ser/Thr protein kinase family, it is the catalytic subunit of a highly conserved protein kinase complex called M phase promoting factor (MPF), which plays a pivotal role in the G2/M phase transition of the eukaryotic cell cycle (Fung and Poon, 2005; Wang et al., 2023). In addition, as early as 2005, Donna E Hansel et al. compared the gene expression profiles of normal esophageal epithelial cell lines and esophageal adenocarcinoma cell lines and found that *CDK1* was significantly upregulated in esophageal adenocarcinoma and was a marker of dysplasia and a drug potential targets (Hansel et al., 2005). In SCLC, clinical studies by Kexin Han et al. have shown that *CDK1* can not only assist in the diagnosis of SCLC, but is also very important in the differential diagnosis of SCLC and NSCLC (Han et al., 2024). The primary function of *EZH2* is to catalyze the methylation of the H3 histone, specifically at residue 27 of the histone, resulting in the formation of H3K27Me3. This process is associated with the repression of transcription of specific genes, including tumor suppressor genes. Additionally, it can form complexes with transcription factors or bind directly to the promoters of target genes, thereby regulating gene transcription (Pasini and Di Croce, 2016; Simon and Lange, 2008). The results of Atsushi Yamada et al. showed that the changes of P53 in esophageal squamous cell carcinoma are related to the abnormal expression of *EZH2*, and the high expression of *EZH2* may promote the progression of esophageal squamous cell carcinoma (Yamada et al., 2011). Xie rui et al.'s study showed that microRNA-30d is a tumor suppressor factor in esophageal squamous cell carcinoma, Luciferase reporter gene assay revealed that *EZH2* is a direct target gene of microRNA-30d. MicroRNA-30d can inhibit the movement of cancer cells by targeting and inhibiting *EZH2* (Xie et al., 2017). In addition, upregulation of *EZH2* expression enhances SCLC progression and radioresistance (Zhai et al., 2020),Eric E Gardner et al. found that in patient-derived xenograft models of SCLC, adding *EZH2* inhibitors to standard chemotherapy regimens can prevent the emergence of acquired drug resistance and enhance the chemotherapy sensitivity of SLCL (Gardner et al., 2017). More studies have confirmed that a variety of cancers are associated with *EZH2* mutations and expression imbalance, and it is an important target for cancer therapy, with 319 targeted drugs currently available (Liu and Yang, 2023). The protein encoded by *KLF4* belongs to the Kruppel family of transcription factors, which play a role in the proliferation and differentiation of epithelial cells. Additionally, this protein is involved in regulating the G1 to S transition of the cell cycle following DNA damage, acting as a mediator for the p53 protein (Wei et al., 2006). *KLF4* plays a significant role in tumor development; however, its function in specific cancer types is not uniform. For instance, it acts as a tumor suppressor in gastrointestinal and skin squamous cell carcinoma (Leng et al., 2020), but as a tumor promoter in skin melanoma and breast cancer (Hu et al., 2015; Li et al., 2012). Guo Zhang et al. demonstrated through CHIP experiments that *KLF4* binds to the promoter of surviving in esophageal squamous cell carcinoma and inhibits its activity, which can induce apoptosis of cancer cells (Zhang et al., 2009), *KLF4* expression is also altered in neuroendocrine lung tumors, and its downregulation may be associated with invasiveness (Naranjo Gómez et al., 2014). Results of this research showed that *KLF4* was significantly downregulated in SCCE and act as a tumor suppressor. *BIRC5* belongs to the inhibitor of apoptosis (IAP) gene family, which encodes negatively regulated proteins that prevent apoptosis (Unruhe et al., 2016). Bioinformatics analysis has revealed that *BIRC5* is highly expressed in many cancers, promotes tumor progression and is strongly associated with poor prognosis (Ye et al., 2022). Interestingly, *BIRC5* has been shown to be overexpressed in esophageal adenocarcinoma and is one of the autophagy-related genes, which may serve as a biomarker for the diagnosis and prognosis of esophageal adenocarcinoma (Zhu et al., 2020). In SCLC, *BIRC5* can also be used as a new therapeutic target. Yang Yunchu et al. knocked down *BIRC5*, which significantly inhibited cell growth and migration and induced cell apoptosis (Yunchu et al., 2024). The protein encoded by *FOXM1* is a transcriptional activator involved in cell proliferation, it undergoes phosphorylation during the M phase of the cell cycle and regulates the expression of multiple cell cycle-related genes (Laoukili et al., 2005). Research by L Gai et al. found that the protein expression of *FOXM1* is significantly upregulated in esophageal squamous cell carcinoma, knockdown the *FOXM1* can inhibit the migration and invasion ability of cancer cells, and is significantly positively correlated with lymph node metastasis, clinical stage, and tumor infiltration depth (Gai et al., 2016). Epigenomic analysis by Benjamin Ziman et al. found that *FOXM1* plays an important role in the development of esophageal adenocarcinoma and regulates anti-tumor immune response in esophageal adenocarcinoma (Ziman et al., 2024). *FOXM1* is also upregulated in SCLC and is significantly associated with poor prognosis. Knockdown of *FOXM1* induces apoptosis of SCLC cells and enhances chemotherapy sensitivity, and can be used as a prognostic biomarker (Liang et al., 2021). A large number of studies have shown that *FOXM1* can promote the progression and metastasis of various cancers, as well as induce chemotherapy resistance, and has become a target for the treatment of various cancers (Khan et al., 2023). *UBE2C* is a member of the E2 ubiquitin ligase family, the encoded protein is required to disrupt mitotic cell cycle proteins and cell cycle progression. It has been reported to have pro-carcinogenic effects in esophageal, lung, colorectal, ovarian and liver cancer (Bavi et al., 2011; Pagano, 1997). Previous studies have shown that *UBE2C* is highly expressed in esophageal squamous cell carcinoma and plays different roles in different stages of esophageal squamous cell carcinoma, mainly affecting the biological function of esophageal squamous cell carcinoma through synergistic effects with *CDK1*, *PTTG1* and *SKP2* (Li et al., 2021b; Palumbo et al., 2016), its upstream *ECRG4* downregulates the expression of *UBE2C* in ESCC cells through NF-κB signal transduction, and *UBE2C* is involved in anti-proliferation and pro-apoptosis functions (Li et al., 2018). High expression of *UBE2C* and sensitivity of tumor cells after silencing *UBE2C* were also detected in esophageal adenocarcinoma (Lin et al., 2006). Through bioinformatics and identification of patient tissue samples, it was found that *UBE2C* expression was also upregulated in SCLC and was identified as a hub gene (Chen et al., 2020). In addition, studies have shown that *UBE2C* is a transcriptional target of *FOXM1*, and the two are closely related (Nicolau-Neto et al., 2018). The protein encoded by *AURKA* is a cell cycle-regulated kinase that promotes the formation and/or stabilization of microtubules at both poles of the spindle during chromosome segregation. It may contribute to the development of tumors by participating in the proliferation of cancer cells, epithelial-mesenchymal transition (EMT), apoptosis and cancer stem cell self-renewal (Du et al., 2021). Previous studies have shown that *AURKA* can cooperate with *TPX2* to regulate the progression of esophageal squamous cell carcinoma through EGFR/PI3K/Akt pathways (Du et al., 2023; Du et al., 2020), it can also regulate the expression and phosphorylation level of *JAK2* to promote the activity of *STAT3*, this mechanism plays an important role in gastric cancer and esophageal cancer (Katsha et al., 2014). In addition, knockdown of *AURKA* can enhance the ferroptosis effect of esophageal squamous cell carcinoma cells (Mi et al., 2024). *AURKA* has been shown to be an important factor in the development of SCLC, Knockdown of *AURKA* can induce SCLC cell cycle arrest and apoptosis, thereby inhibiting the progression of SCLC (Lu et al., 2014). Yixiang Li et al. found that some SCLCs are highly sensitive to *AURKA* inhibitors, but the persistence is poor, the combination of specific *AURKA* inhibitors and *PD-L1* showed a lasting inhibitory effect (Li et al., 2023). In summary, the seven genes found in our study were not only identified as hub genes in this study, but also played an important role in similar esophageal squamous cell carcinoma, esophageal adenocarcinoma, and small cell lung cancer, which also shows that the conclusions drawn in this study have a certain degree of reliability. However, they must play different roles in different types of cancer, and clinical analysis can be performed to further study whether they can be used as differential diagnostic markers for several similar types of cancer. There is no overlap between the hub genes identified in this study and those found in other bioinformatics studies on SCCE, as the selection of hub genes would differ when using different analysis processes.

Using hub genes and DEGs for prediction in the database, a total of 38 drug candidates were screened, including commonly used cancer chemotherapy drugs such as etoposide, cisplatin, fluorouracil, and cladribine. The 38 small molecule compounds were molecularly docked with seven hub genes, and the results showed that most drugs had good binding affinity with *AURKA, CDK1* and *EZH2* proteins, and had the potential to become inhibitors of the corresponding targets, thereby playing a role in the treatment of SCCE. Hub genes obtained in this study have been previously demonstrated by a large number of literature to be oncogenes or tumor suppressor genes, and they play an important role in various cancers. However, this is the first time that they have been reported in SCCE, and may be related to certain mechanisms of SCCE pathogenesis. Later, more correlation analysis of clinical pathological characteristics may further reveal more mechanisms of SCCE pathogenesis, and can also verify the value of these genes as SCCE diagnosis or prognosis. The anti-tumor drugs predicted based on these genes were also reported for the first time in SCCE. Clinically, there are very limited drugs used to treat SCCE, and most chemotherapy drugs have a certain degree of drug resistance in patients. Therefore, some new drugs are urgently needed, and completely new drugs take a long time and consume a lot of resources from research and development to final clinical application. Our research provides drugs that have not yet been used in SCCE but have been used in other tumors clinically, studies have shown that they have great potential for treating SCCE. This study further explored the specific sites of action between drugs and targets, providing the possibility of studying these drugs in the direction of targeted therapy, which may have surprising discoveries. Preclinical and clinical studies on their safety and efficacy may enable them to be used in clinical practice as soon as possible, alleviating the current clinical dilemma.

However, the dataset used in this study only has three samples, the sample size is small, and due to the rarity of SCCE, it was difficult to experimentally verify the reliability of hub genes. Therefore, whether these drugs are truly effective for SCCE still requires a large number of *in vitro* and *in vivo* experiments to confirm. The present study offers a novel approach to investigating the etiology of rare diseases and identifying prospective pharmaceutical agents.

## 5 Conclusion

In summary, it can be concluded that *AURKA, BIRC5*, *CDK1*, *EZH2*, *FOXM1*, *KLF4* and *UBE2C* may be the hub genes closely associated with the development of SCCE. In addition, crizotinib with *AURKA*, lapatinib with *CDK1*, rucaparib with *EZH2* and *UBE2C* have good binding affinity, suggesting that these drugs may work against the aforementioned targets. *CDK1*, *EZH2*, *AURKA* proteins have good binding ability with several drugs, further suggesting that targeting these three genes to find drugs may be a powerful approach for the treatment of SCCE. However, it is necessary to validate these conclusions through *in vitro* and *in vivo* experiments.

## Data Availability

The original contributions presented in the study are included in the article/Supplementary Material, further inquiries can be directed to the corresponding author.

## Supplementary material

SUPPLEMENTARY FIGURE S1 Prediction of targeted miRNAs and lncRNAs. **(A)** Venn diagram of the predicted miRNAs via miRDB, miRwalk and Targetscan database. **(B)** Venn diagram of predicted miRNAs and OncomiR. **(C)** Venn diagram of the predicted lncRNAs via LncBase and starbase.
