# Supplementary figures and images for: Integrated bioinformatics analysis to explore potential therapeutic targets and drugs for small cell carcinoma of the esophagus
Source: Front Bioinform. 2025 Jan 28;5:1495052. doi: 10.3389/fbinf.2025.1495052 (PMC11810980; doi:10.3389/fbinf.2025.1495052)

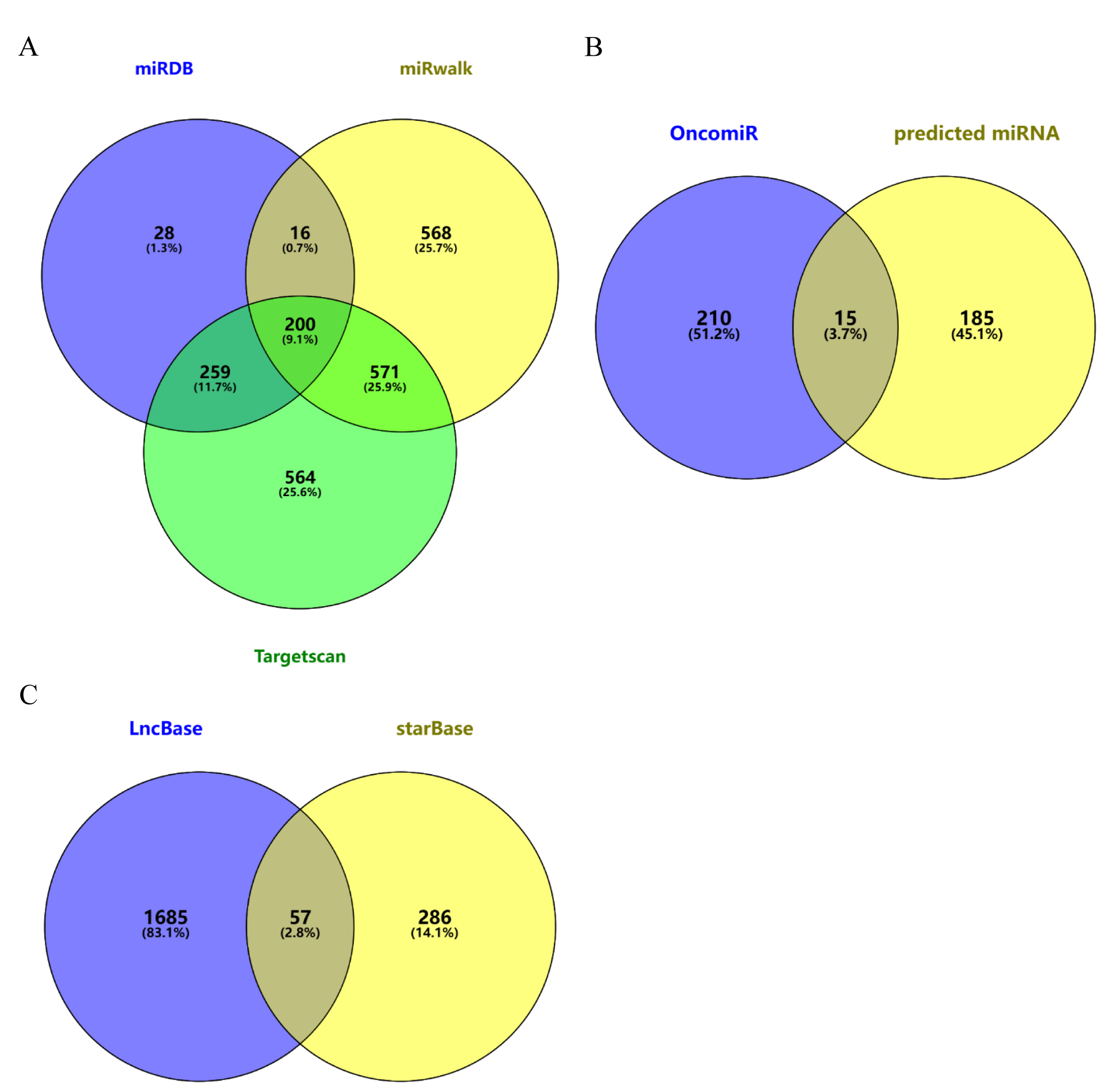

Supplement: Supplementary file 1 [file Image1.tif]
